# Genome-Wide Identification and Expression Analysis of the *Fructose 1,6-Bisphosphate Aldolase* (*FBA*) Gene Family Members in Seashore Paspalum in Response to Cadmium Stress

**DOI:** 10.3390/cimb48060563

**Published:** 2026-05-28

**Authors:** Yu Liu, Hao Liang, Xiaxiang Zhang, Qiang Zhang, Nanqing Liu

**Affiliations:** 1 College of Landscape Architecture, Jiangsu Vocational College of Agriculture and Forestry, Zhenjiang 212400, China; 2 College of Agro-Grassland Science, Nanjing Agricultural University, Nanjing 210014, China

**Keywords:** *Paspalum vaginatum*, gene family identification, abiotic stresses, protein structure, qRT-PCR, molecular breeding

## Abstract

The *fructose 1,6-bisphosphate aldolase* (*FBA*) gene family plays crucial roles in plant energy metabolism, growth, development, and abiotic stress responses, as it modulates antioxidant synthesis and soluble sugar accumulation to enhance plant cadmium tolerance. Seashore paspalum (*Paspalum vaginatum* Sw.), a halophytic perennial C_4_ turfgrass renowned for its exceptional cadmium tolerance, is ideal for phytoremediation of cadmium-contaminated soil. *FBA* family genes have been identified in several grass species, such as maize, rice, and wheat, but systematic investigations into *FBA* family genes and their functions in seashore paspalum remain scarce. In this study, seven class I *FBAs* (named as *PvFBA1*–*PvFBA7*) and one class II *FBA* (named as *PvFBA8*) in seashore paspalum were identified. The physicochemical properties, evolutionary relationships, gene structures, conserved domains, protein structures, *cis*-acting regulatory elements, chromosomal localizations, and collinearity relationships of eight *PvFBAs* were analyzed. These analyses suggested that *PvFBA* genes had highly conserved domains and belonged to ultra-conserved core genes. Expression pattern analysis indicated that the *PvFBA* gene family was dynamically responsive to cadmium stress. *PvFBA6* and *PvFBA7* were highly expressed in leaves, whereas *PvFBA1* and *PvFBA3* showed almost no expression. The RT-qPCR results suggested that the expression levels of *PvFBA5* and *PvFBA6* were highly consistent with the FPKM value trends analyzed in the transcriptomic data. Collectively, this study not only provides a theoretical foundation for the understanding of the evolution of the *PvFBA* gene family but also offers potential candidate genes for enhancing cadmium stress tolerance in plants.

## 1. Introduction

Fructose-1,6-bisphosphate aldolase (FBA; EC 4.1.2.13) is an essential enzyme in glycometabolism. FBAs can be divided into two classes based on different catalytic mechanisms and the evolutionary origins of species. Class I FBAs are mainly distributed in plants and animals, while class II FBAs are mainly found in microorganisms and a few algae and plant species. In plants, two distinct isozyme forms are recognized: cytoplasmic and chloroplast-localized [1]. The cytoplasmic type is a key enzyme in the sucrose biosynthetic and glycolytic/gluconeogenesis pathways, while the chloroplast type is a critical enzyme in the Calvin–Benson cycle [2]. FBA not only catalyzes the reversible reaction of fructose-1,6-diphosphate (FBP) converting into dihydroxyacetone phosphate (DHAP) and glyceraldehyde-3-phosphate (G3P) but also mediates the reversible reaction of erythrose-4-phosphate (E4P) and DHAP to form sedoheptulose-1,7-bisphosphate (SuBP) [3].

FBA plays a significant role in coordinating intracellular metabolic integration and energy flow within cells, as well as in regulating growth and development processes [2]. In *Arabidopsis thaliana*, overexpression of *NtFBA* enhances the activity of aldolase, growth rate and biomass [4], while *AtFBA1*, *AtFBA2* and *AtFBA3* are involved in photosynthetic or non-photosynthetic metabolism [5]. *FBA* genes are also involved in the response regulation of various abiotic stresses, mainly including salinity [6], cold [7,8,9], cadmium [10], heat [11], and suboptimal temperature [12]. These findings highlight that *FBA* genes are promising targets for plant genetic engineering aimed at improving crop yield, quality and stress resistance.

Poaceae, also called the grass family, is one of the most geographically widespread, economically significant and ecologically dominant plant families globally [13]. Seashore paspalum (*Paspalum vaginatum* Sw.), a halophytic perennial C_4_ turfgrass, is native to the tropical and subtropical Americas. This species, together with maize (*Zea mays* L.) and sorghum (*Sorghum bicolor* L.) belongs to the Poaceae family and shares a close phylogenetic relationship [14]. It is widely utilized in animal forage, commercial and residential landscaping, golf courses, sports fields, erosion control, wetland restorations, and site reclamation on oil and gas well sites [15,16]. It is highly tolerant of various environmental stresses, making it an ideal choice for environmental turfgrass applications [17,18,19,20]. Although *FBA* gene families have been extensively characterized in *Arabidopsis* [21], tomato (*Solanum lycopersicum* L.) [12], wheat (*Triticum aestivum* L.) [11], cucumber (*Cucumis sativus* L.) [22], and sweet potato (*Ipomoea batatas* L.) [23], limited information exists regarding this gene family in the stress-tolerant non-model plant, seashore paspalum. This study aims to fill this knowledge gap by conducting a comprehensive genome-wide analysis of the *FBA* gene family in seashore paspalum and investigating its expression patterns under cadmium stress.

In this study, eight *FBA* genes were identified in seashore paspalum through the comprehensive analysis of the *FBA* gene family and the recently published high-quality genome assembly [14]. The analysis encompassed several aspects, including evolutionary relationships, gene structures, conserved domains, and *cis*-acting regulatory elements. Furthermore, we determined the expression patterns of *PvFBA* genes in response to cadmium stress. These findings lay a critical foundation for understanding the molecular regulatory mechanism of the *PvFBA* gene family under cadmium stress and subsequently are helpful in the development of grass breeding and environmental remediation.

## 2. Materials and Methods

### 2.1. Genome-Wide Identification of the FBA Gene Family in Seashore Paspalum

The whole-genome sequence of seashore paspalum was downloaded from the Phytozome database (https://phytozome-next.jgi.doe.gov/info/Pvaginatum_v3_1/, accessed on 12 November 2025) to establish a local database. The genomic data of the maize and rice (*Oryza sativa* L.) *FBA* gene family were downloaded from the Maize Genetics and Genomics Database (MaizeGDB, https://www.maizegdb.org/, accessed on 12 November 2025) and the Rice Genome Annotation Project (RGAP, https://rice.uga.edu/, accessed on 12 November 2025). The protein sequences of *Arabidopsis*, potato, wheat, and tomato *FBA* gene family were sourced from the TAIR database (http://www.arabidopsis.org, accessed on 20 November 2025), and the Ensemble database (http://plants.ensembl.org/index.html, accessed on 20 November 2025), reserved for subsequent analysis. Using the amino acid sequences of maize and rice FBA proteins as queries, an algorithm based on BLASTP (E-value < 1 × 10^−10^ and other parameters as default values) was used in the protein databases of seashore paspalum using the TBtools-II software (v2.376, https://github.com/CJ-Chen/TBtools-II/releases, accessed on 12 November 2025) [24]. The Hidden Markov Model (HMM) of the glycolytic (PF00274.26) and fructose–bisphosphate aldolase class II (PF01116.27) domains was extracted from the InterPro database (https://www.ebi.ac.uk/interpro/, accessed on 12 November 2025) to identify all PvFBA proteins using the HMMER SEARCH (v3.3.2) program (E-value < 1 × 10^−5^ and other parameters as default values, http://www.hmmer.org/, accessed on 12 November 2025) [25], and then, redundant sequences without completed glycolytic or fructose–bisphosphate aldolase class II domains (coverage < 70%) were manually deleted by the Simple Modular Architecture Research Tool (SMART) (https://smart.embl.de/smart/change_mode.cgi, accessed on 20 November 2025) and the NCBI Conserved Domain database (CDD) (https://www.ncbi.nlm.nih.gov/Structure/cdd/wrpsb.cgi, accessed on 20 November 2025). Finally, a total of eight predicted PvFBA protein sequences were obtained.

### 2.2. Prediction of Physicochemical Properties of PvFBA Proteins

The key physicochemical properties of the identified PvFBA proteins were predicted, including length, isoelectric point (pI), molecular weight (MW), and grand average of hydropathicity (GRAVY), using the Expert Protein Analysis System (ExPASy) server (https://www.expasy.org/, accessed on 21 November 2025). Concurrently, the putative subcellular localization of eight PvFBA proteins was analyzed using the WoLF PSORT online tool (https://wolfpsort.hgc.jp/, accessed on 21 November 2025).

### 2.3. Phylogenetic Analysis of PvFBA Proteins

The ClustalW software (v2.1) [26] was used to analyze eight PvFBA protein sequences of seashore paspalum, ten ZmFBA protein sequences of maize (relative of seashore paspalum), and eight OsFBA protein sequences of rice (relative of seashore paspalum) with default parameters. The phylogenetic tree of FBA proteins from seashore paspalum, maize, rice, *Arabidopsis*, potato, wheat, and tomato was constructed using the unrooted neighbor-joining method in the MEGA X software (v10.2.6 https://megasoftware.net/, accessed on 20 November 2025) [27] with a bootstrap value of 1000 replicates, default parameters and the Poisson correction model, and the tree was further refined by the Interactive Tree Of Life (ITOL) online tool (https://itol.embl.de/, accessed on 20 November 2025).

### 2.4. Gene Structure, Conserved Motif and Promoter Analysis

The coding sequence (CDS) and its corresponding genomic sequence of *PvFBA* family members were obtained from the Phytozome database and visualized by the TBtools-II software (v2.376) [24]. The conserved domains and motifs of PvFBAs were analyzed using the CD-search tool (https://www.ncbi.nlm.nih.gov/Structure/bwrpsb/bwrpsb.cgi/, accessed on 21 November 2025) (all other parameters being default) and the MEME (v5.5.9) suite (https://meme-suite.org/meme/, accessed on 21 November 2025) (motif length ranging from 6 to 60 amino acid residues, maximum motif number set to 20, with other parameters as default values). The 2000 bp sequences upstream of the initiation codon, as a hypothetical promoter, were extracted from the Phytozome database and then submitted to the PlantCare online tool (https://bioinformatics.psb.ugent.be/webtools/plantcare/html/, accessed on 21 November 2025) [28] to predict *cis*-acting regulatory elements. Visualizations of the *cis*-acting regulatory elements were drawn by using the TBtools-II software (v2.376) [24].

### 2.5. Structure Prediction of PvFBA Proteins

The secondary structures of eight PvFBA proteins were predicted by using the Network Protein Sequence Analysis (SOPMA v20251015, https://npsa.lyon.inserm.fr/, accessed on 6 January 2026) online software [29]. AlphaFold2, an artificial intelligence (AI) program, was used to predict protein tertiary structures of eight PvFBA proteins [30]. Eight models of PvFBA proteins can be downloaded from the Supplementary Materials (Model S1). The PyMOL software (v3.1.6.1, https://pymol.org/, accessed on 10 January 2026), a molecular visualization and analysis software for 3D structures, was used for visual analytics of the prediction structures of AlphaFold2 [30].

### 2.6. Chromosomal Distributions and Collinearity Analysis

MapGene2Chrome (MG2C, v2.1, http://mg2c.iask.in/mg2c_v2.1/, accessed on 2 November 2025), a tool used to draw gene physical maps, was used to draft a chromosomal location map of the *PvFBA* genes based on the genome annotation files of seashore paspalum [31]. The synteny relationships of the *FBA* genes within and among seashore paspalum, maize, and rice were analyzed by TBtools-II software (v2.376) [24] using the One Step MCScanX method [32], with an E-value parameter of <1 × 10^−5^.

### 2.7. Gene Expression Pattern Analysis

To analyze the expression levels of the *PvFBA* genes, the published transcriptome data of seashore paspalum [33] were downloaded for the calculation. Fragments per kilobase of exon per million fragments mapped (FPKM) values were obtained from the transcriptome data and normalized via log2 transformation. The heatmap was generated by the TBtools-II software (v2.376) [24].

### 2.8. Cadmium Stress Treatment of Seashore Paspalum

Erect stems of seashore paspalum (cultivar ‘Sea Spray’) were cut and hydroponically cultured in black plastic containers (31 cm length, 28.5 cm width, and 18 cm depth) filled with Hoagland’s nutrient solution (pH  =  6.0, renewed every 7 days; the solution contained 5.0 mM Ca(NO_3_)_2_·4H_2_O, 5.0 mM KNO_3_, 2 mM MgSO_4_·7H_2_O, and 1.0 mM KH_2_PO_4_, 46.2 μM H_3_BO_3_, 9.1 μM MnCl_2_·4H_2_O, 0.76 μM ZnSO_4_·7H_2_O, 0.32 μM CuSO_4_·5H_2_O, 0.10 μM Na_2_MoO_4_·2H_2_O, and 53.7 μM Fe-EDTA) [34] and then maintained in a controlled growth chamber (MT8070iE, Xubang, Jinan, China) with growing conditions maintained at 30/25 °C (day/night), 70% relative humidity, and a 14/10-h photoperiod with photosynthetically active radiation of 650 μmol m^−2^ s^−1^ for three weeks. Three-week-old seashore paspalum was utilized for the cadmium treatment of 300 μM CdCl_2_. Leaf, stem, and root samples (0.1 g each) were collected at five time points (0, 1, 4, 12, and 24 h) under cadmium treatment and stored at −80 °C for further analysis.

### 2.9. RNA Extraction and RT-qPCR Analysis

Total RNA was extracted using the E.Z.N.A.^®^ Plant RNA Kit (OMEGA Bio-Tek, Norcross, GA, USA) according to the manufacturer’s instructions and reverse-transcribed into cDNA with the PrimeScript™ RT reagent Kit with gDNA Eraser (Takara, Kusatsu, Japan). The RT-qPCR analysis of target genes was conducted using a LightCycler 480 II Instrument (Roche Diagnostics, Rotkreuz, Switzerland). The RT-qPCR reaction volume was 20 μL, containing 10 μL of 2× SYBR Green Master Mix (Roche), 1 μL of forward primer, 1 μL of reverse primer, 2 μL of cDNA template, and 6 μL of double-distilled water. The reaction conditions were set as follows: initial denaturation at 95 °C for 10 min, followed by 40 cycles of 95 °C for 15 s, 60 °C for 15 s, and 72 °C for 20 s. *PvU2AF* [35] (chosen for its expression stability under cadmium stress) was used as the internal reference gene for seashore paspalum’s response to cadmium stress and was employed to normalize the expression levels of each gene. All RT-qPCR primers are listed in Supplementary Table S1. The specificity of all primers was confirmed by melting curve analysis of qRT-PCR amplicons, each yielding a single peak (Figure S1). All RT-qPCR experiments were performed using three independent biological replicates, and each biological sample had three technical replicates. The expression levels of target genes were calculated employing the 2^−ΔΔCT^ method [36].

### 2.10. Statistical Analysis

The statistical analyses of all data were subjected to one-way analysis of variance (ANOVA) using the SPSS software (v22, SPSS Inc., Chicago, IL, USA). The statistical significance of the difference was assessed using Fisher’s protected LSD test (*p* < 0.05). Data shown are mean values ± standard error (SE) of three independent biological replicates and three technical replicates. The GraphPad Prism software (v10.1.2, GraphPad Software LLC, Boston, MA, USA) was used to draw all figures.

## 3. Results

### 3.1. Identification and Protein Characterization of the PvFBA Gene Family

To explore the *PvFBA* gene family, a total of eight *PvFBA* genes (named *PvFBA1*–*PvFBA8*) were identified by whole-genome screening of seashore paspalum using the *FBA* genes of rice [37] and maize [38]. Specific details of these *PvFBA* genes, including gene name, gene ID, chromosome location, protein length, pI, MW, GRAVY, and predicted subcellular localization, were analyzed (Table 1). The protein length of eight PvFBA proteins ranged from 359 amino acids (aa; PvFBA5) to 1376 aa (PvFBA8). The pI values ranged from 5.93 for PvFBA5 to 9.04 for PvFBA4, with five PvFBA proteins having acidic properties. The MW ranged from 37992.35 Da (PvFBA5) to 147793.06 Da (PvFBA8). The GRAVY indicates that all members of the PvFBA family (except PvFBA8) were hydrophilic proteins, as they exhibited negative hydrophilicity indices. Eight PvFBA proteins were located in two cellular compartments: PvFBA1–PvFBA4 were in the chloroplast and were thus named CpFBAs, whereas PvFBA5–PvFBA8 were localized in the cytoplasm and are hereafter designated as cFBAs (Table 1).

### 3.2. Classification and Phylogenetic Analysis of PvFBAs

**Figure 1:**
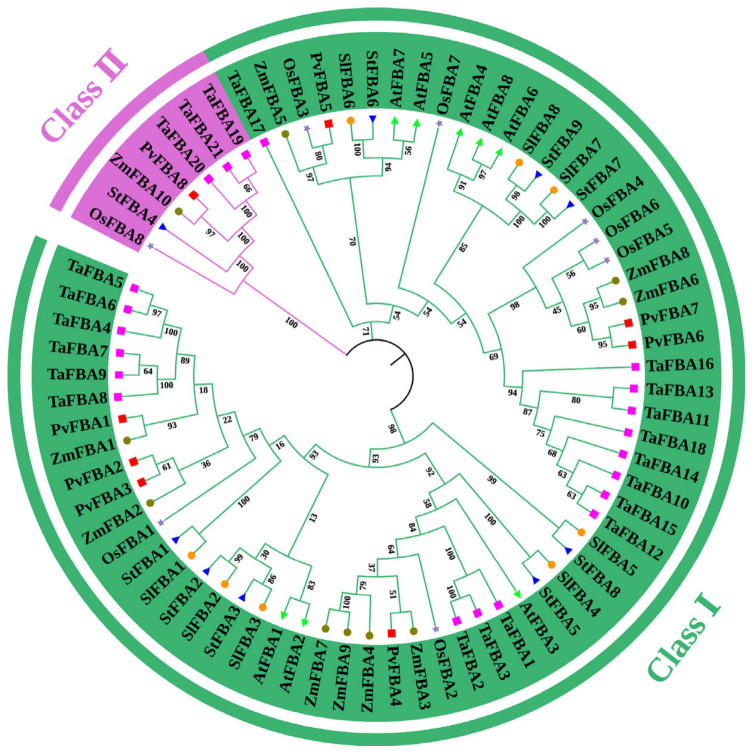
Evolutionary relationships of the *FBA* gene family in seashore paspalum, maize, rice, *Arabidopsis*, potato, wheat, and tomato. Based on the neighbor-joining method with the MEGA X software, a total of 72 FBAs were classified into two subfamilies (classes I and II, denoted with green and purple, respectively). The red squares represented eight PvFBAs in seashore paspalum. The olive circles represent ten ZmFBAs in maize. The violet stars represent eight OsFBAs in rice. The green triangles represent eight AtFBAs in *Arabidopsis*. The blue triangles represent nine StFBAs in potato. The fuchsia squares represent twenty-one TaFBAs in wheat. The orange circles represent eight SlFBAs in tomato.

To investigate the evolutionary relationships among the FBA proteins of plants, a phylogenetic tree was constructed using multiple sequence alignment (Figure 1). The phylogenetic tree included eight PvFBAs, ten ZmFBAs, eight OsFBAs, eight AtFBAs, nine StFBAs, twenty-one TaFBAs, and eight SlFBAs (Table S2), which were divided into two subfamilies (classes I and II) based on the evolutionary distance. Class I included sixty-five members, while class II contained only seven members. It is worth noting that three members of class II come from seashore paspalum, maize, potato, and rice (Figure 1). Therefore, seven *FBA* genes, *PvFBA8*, *ZmFBA10*, *OsFBA8*, *StFBA4*, and *TaFBA19*-*TaFBA21*, had sequence differences from other *FBA* genes and might have functional differences.

### 3.3. Gene Structure and Conserved Domain Analysis of PvFBAs

**Figure 2:**
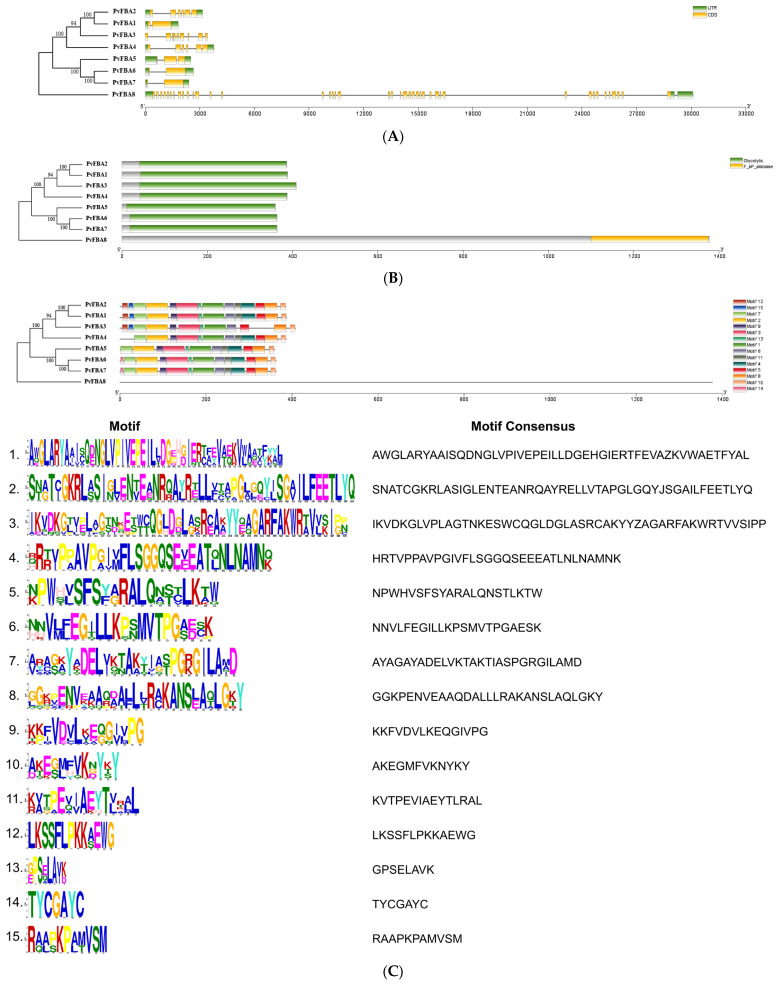
Exon–intron structures, gene domains, and conserved motifs of *PvFBA* genes. (**A**) Gene structure analysis of *PvFBA* genes. The untranslated region (UTR) and CDS are shown in green and yellow respectively. (**B**) Conserved domain analysis of PvFBA proteins. Glycolytic (PF00274) and fructose–bisphosphate aldolase domains (F_bP_aldolase; PF01116) are shown in green and yellow respectively. (**C**) Conserved motif analysis of PvFBA proteins. Fifteen putative motifs are indicated in different colored boxes. The information on the motifs is listed at the bottom.

To understand the gene evolution and potential functions of the *PvFBA* gene family, their gene structures and conserved domains were analyzed (Figure 2). For the seven class I *PvFBA* genes, the number of exons ranged from two to nine, with the fewest exons found in *PvFBA1*, *PvFBA6*, and *PvFBA7* and the most in *PvFBA3*. Class II *PvFBA8* contained 42 exons, which is significantly more than class I *PvFBA* members. This suggested that *PvFBA8* might have a more complex structure, potentially including functional domains or regulatory elements. Notably, *PvFBA3* had no UTRs, while the other seven *PvFBAs* had two UTRs (Figure 2A). The conserved domain analysis of the PvFBA protein sequences showed that, except for PvFBA8, the other PvFBA proteins contained a glycolytic domain located at the protein C-terminus (carboxyl terminus). However, *PvFBA8* contained a fructose–bisphosphate aldolase domain (F_bP_aldolase) located at the protein C-terminus (Figure 2B). A total of fifteen motifs (named motif1–motif15) were identified, ranging in length from 9 to 30 aa, as the most prevalent structural elements within the *PvFBA* gene family. Ten motifs, motifs 1, 2, 3, 5, 6, 7, 8, 9, 10, and 13, were the most conserved and widely present in seven class I *PvFBA* genes. Although motifs 1, 2, and 3 were longer and contained 30 aa, they appeared only in adjacent branches of the phylogenetic tree. Notably, *PvFBA8* lacked these motifs but still belonged to the *PvFBA* gene family (Figure 2C). The differences in the gene structure or conserved motifs of the *PvFBA* genes might be related to the complex biological functions.

### 3.4. Tertiary Structure of the PvFBA Proteins

**Figure 3:**
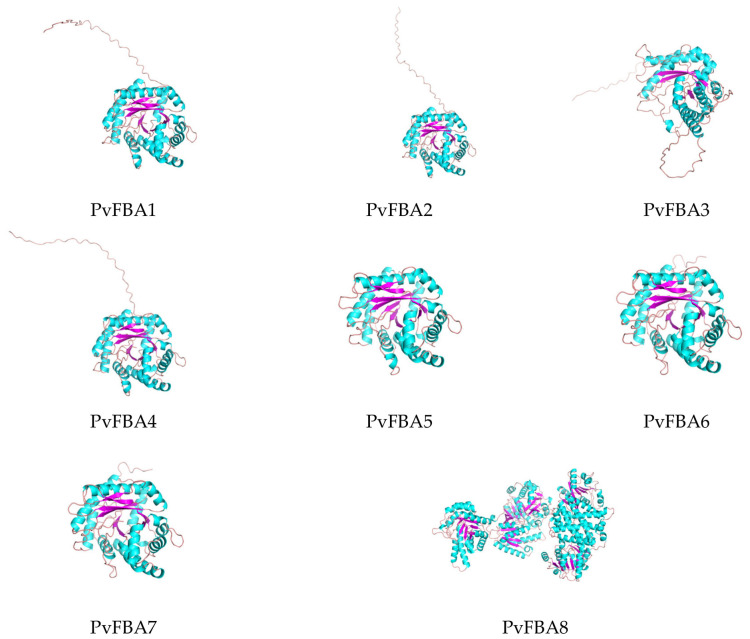
Predicted tertiary structures of PvFBA proteins. Helices, sheets, and loops are represented by cyan, magenta, and salmon, respectively.

The structure of proteins is crucial to their functions. Secondary structure involves folding the amino acid chain into repeating structures, such as α-helices and β-turns. The tertiary structure determines functional specificity and cellular stability [39]. Analyses of tertiary structures revealed that PvFBA mainly consists of α-helices, extended strands, β-turns, and random coils. The proportion of α-helices was the highest (ranging from 46.58% to 53.61%), followed by random coils (ranging from 30.08% to 34.31%), while extended strands and β-turns accounted for a lower proportion (Table S4). Analysis of the predicted tertiary structures of the PvFBA proteins revealed that there were significant differences in the protein structures of classes I and II. The three-dimensional conformations of seven class I PvFBA proteins were comparable. Six class I PvFBA proteins, PvFBA1, PvFBA2, PvFBA4, PvFBA5, PvFBA6, and PvFBA7, were predicted by AlphaFold2 to have more consistent protein structures. The sole member of the class II, PvFBA8, had a longer polypeptide chain and a more complex protein tertiary structure (Figure 3), suggesting that its biological function was different from other PvFBAs and may be involved in more complex biological processes. It is also noteworthy that all predicted local distance difference test (pLDDT) scores for the eight PvFBA proteins exceeded 70 (Table S5 and Figure S2), indicating reliable prediction at the individual domain level.

### 3.5. Cis-Acting Regulatory Elements Analysis in the PvFBA Gene Family

**Figure 4:**
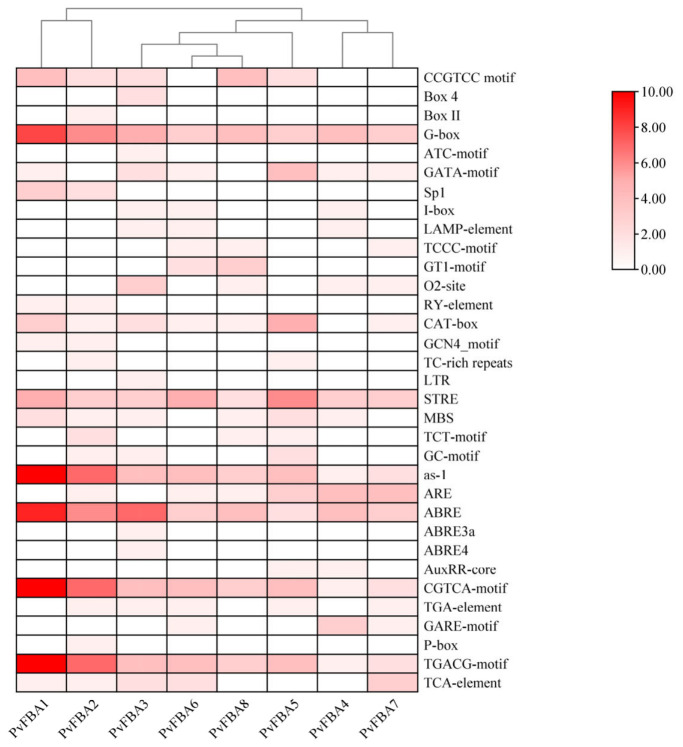
*Cis*-acting regulatory element analysis in the promoters of *PvFBA* genes. The red color depth of the square represents the number of *cis*-acting regulatory elements in the promoters of *PvFBAs*.

To clarify the potential function of *PvFBAs*, a 2000 bp sequence upstream of the predicted initiation codon for eight *PvFBA* genes was analyzed for putative *cis*-acting regulatory elements using the PlantCARE database. Thirty-seven putative *cis*-acting regulatory elements were identified in the *PvFBA* promoters (Table S3). *PvFBA2* and *PvFBA3* promoters had the most *cis*-acting regulatory elements (twenty-three), whereas *PvFBA8* promoters had the fewest *cis*-acting regulatory elements (fourteen). Twelve phytohormone-associated elements were detected, associated with the plant’s response to auxin (as-1), abscisic acid responsiveness (ABRE), abscisic acid response element (ABRE3a and ABRE4), auxin responsiveness (AuxRR-core), *cis*-acting regulatory element involved in the MeJA-responsiveness (TGACG-motif), auxin-responsive element (TGA-element), gibberellin-responsive element (GARE-motif), MeJA-responsiveness (CGTCA-motif), ethylene-responsive element (ERE), salicylic acid responsiveness (TCA-element), and gibberellin-responsive element (P-box). Ten light-responsive elements were found in the promoters of *PvFBA* genes, including part of a conserved DNA module involved in light-responsiveness (Box 4), part of a light-responsive element (GATA-motif, LAMP-element, and TCCC-motif), a light-responsive element (G-box, Sp1, and GT1-motif), light-responsiveness (ATC-motif), part of a light-responsive module (AE-box), and an MYB binding site involved in light-responsiveness (MRE). Eight stress-responsive elements were identified, including low-temperature responsiveness (LTR), drought-inducibility (MBS), enhancer-like element involved in anoxic-specific inducibility (GC-motif), stress-responsive element (STRE), dehydration-responsive element (DRE and DRE core), and defense and stress responsiveness (TC-rich repeats). Seven growth and development-related elements were observed, including a *cis*-acting regulatory element related to meristem-specific activation (CCGTCC motif and CCGTCC-box), zein metabolism regulation (O2-site), meristem expression (CAT-box), a *cis*-acting regulatory element involved in endosperm expression (GCN4_motif), an MYBHv1 binding site (CCAAT-box), and a root-specific *cis*-acting regulatory element (motif I). Overall, six core/binding elements, specifically G-box, as-1, STRE, ABRE, the TGACG-motif, and the CGTCA-motif, were ubiquitously present across the promoter region of all *PvFBA* genes (Figure 4). These results indicated that *PvFBA* genes were involved in light-responsiveness, hormone-responsiveness, stress adaptation, and growth/development regulation. In conclusion, these analyses indicated that there were varying degrees of differences in the type and number of *cis*-acting regulatory elements among *PvFBA* genes, and the biological functions of these elements required further experimental research.

### 3.6. Chromosomal Distributions and Collinearity Analysis of the FBA Gene Family

**Figure 5:**
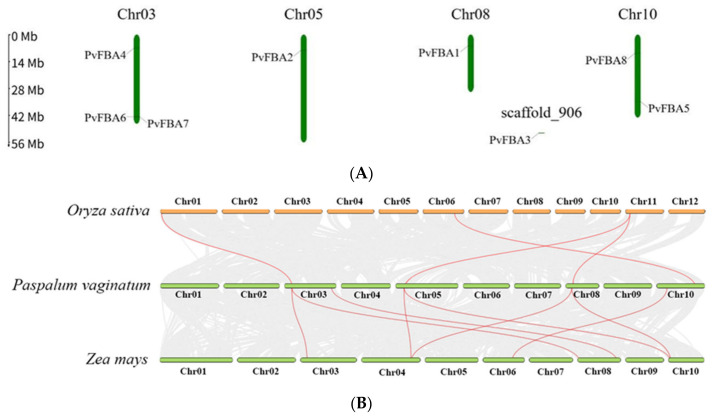
Whole-genome chromosomal distributions and collinearity analysis of the *FBA* genes. (**A**) Chromosomal locations of *PvFBA* genes. (**B**) Collinearity of *PvFBA* genes with those of *FBA* genes from maize and rice. The gray lines between seashore paspalum and maize/rice represent collinearity in wide regions of the whole genomes, while the red lines indicate the duplicated relationships of *FBA* genes.

All *PvFBA* genes except *PvFBA3*, which was located on unmapped scaffolds, were unevenly distributed on four chromosomes. There were three genes (*PvFBA4*, *PvFBA6*, and *PvFBA7*) on chromosome 03, two genes (*PvFBA5* and *PvFBA7*) on chromosome 10, and one gene on each of the other two chromosomes (Figure 5A). Collinearity analysis among seashore paspalum, maize, and rice uncovered greater homology between seashore paspalum and maize *FBA* genes compared to rice, with eight orthologous gene pairs identified in maize and only four in rice (Figure 5). These results showed that the *PvFBA* gene family was more closely related to that of maize than to that of rice.

### 3.7. Expression Patterns of PvFBA Genes in Leaves Under Cadmium Stress Treatment

**Figure 6:**
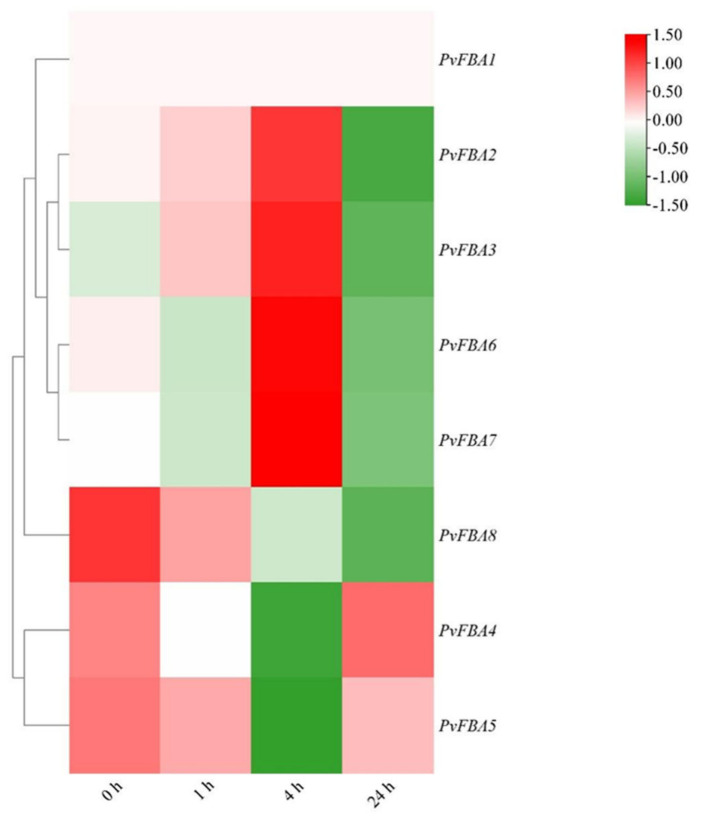
Expression patterns of *PvFBA* genes in leaves under cadmium stress treatment. The red color depth of the square represents the high expression level of *PvFBA* genes, while the green color of the square signifies the low expression level of *PvFBA* genes.

To further analyze the biological functions of *PvFBA* genes under different environmental conditions, the transcriptome data of seashore paspalum were downloaded from the NCBI SRA database [33]. The expression patterns of the *PvFBA* gene family under cadmium stress were analyzed, indicating that the expression levels of most *PvFBA* genes changed to varying degrees in response to cadmium stress. In the 1-h treatment, five *PvFBA* genes (*PvFBA4*, *PvFBA5*, *PvFBA6*, *PvFBA7*, and *PvFBA8*) showed downregulated expression. The expression levels of *PvFBA2*, *PvFBA3*, *PvFBA6*, and *PvFBA7* peaked in the 4-h treatment; then, all of them showed a decreasing trend. Notably, *PvFBA8* remained consistently downregulated under prolonged cadmium treatment (1, 4, and 24 h), whereas no expression level of *PvFBA1* was detected (Figure 6). These results suggested that *PvFBA* genes might play a specific role in the tolerance mechanisms to cadmium stress.

### 3.8. Validation of Transcriptomic Data with RT-qPCR

**Figure 7:**
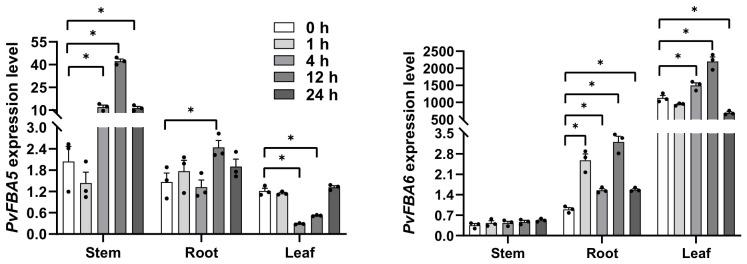
Expression levels of *PvFBA5* and *PvFBA6* detected by RT-qPCR under cadmium stress treatment using 300 μM CdCl_2_. Data shown as the mean values ± SE of three independent biological replicates and three technical replicates. Asterisks indicate significant differences according to Fisher’s protected LSD test (*p* < 0.05).

Two genes, *PvFBA5* and *PvFBA6*, were selected randomly from eight *PvFBA* genes, and their expression levels were analyzed using RT-qPCR to validate the transcriptome data of seashore paspalum in response to cadmium stress. In leaves, the expression level of *PvFBA5* exhibited a significant decrease at 4 h, followed by a gradual increase at 12 and 24 h. In contrast, the expression level of *PvFBA6* peaked at 12 h and then showed a decreasing trend (Figure 7). The RT-qPCR results of *PvFBA5* and *PvFBA6* were consistent with the expression trends from transcriptomic data, validating the reliability of the transcriptomic data results. Moreover, the expression levels of *PvFBA5* and *PvFBA6* were examined in stems and roots under cadmium stress. In roots, cadmium treatment significantly upregulated the expression levels of *PvFBA5* and *PvFBA6*, peaking at 12 h. The expression level of *PvFBA5* showed the most significant induction in stems, with its expression level increasing by 5.8-fold compared to the 0-h condition. These results suggested that *PvFBA5* and *PvFBA6* likely played crucial roles in cadmium stress responses.

## 4. Discussion

Seashore paspalum is a perennial Poaceae species renowned for its exceptional tolerance to cadmium and salinity. It is widely utilized for phytoremediation in cadmium-contaminated areas and as the environmental turfgrass in salinity-affected regions [20,40]. For this species, however, the gene-level basis of cadmium-responsiveness remains incompletely resolved. Because FBA enzymes connect carbon metabolism with stress-related energy and redox processes, characterization of the *PvFBA* family provides a useful entry point for identifying candidate components involved in cadmium response. The *FBA* gene family has been identified in several crops, including maize (ten *ZmFBAs*) [38], rice (eight *OsFBAs*) [37], and wheat (twenty-one *TaFBAs*) [11]. To date, no genome-wide interpretation of the *FBA* family in seashore paspalum has been linked directly with cadmium-responsive expression patterns. In the present study, eight *PvFBA* genes (*PvFBA1*–*PvFBA8*) were identified in the complete genome sequence of seashore paspalum, which were classified into class I and class II members based on their conserved domain architectures (Table 1). Seashore paspalum, like rice, included seven class I FBA members, fewer than the number reported in maize, *Arabidopsis*, potato, wheat, and tomato. This difference may be likely due to the expansion of the FBA gene family in maize following the split between Andropogonea (maize) and Paspaleae (seashore paspalum) approximately 28 million years ago [14]. The collinearity results support this evolutionary explanation, although they do not, by themselves, demonstrate functional divergence among the duplicated genes.

The physicochemical properties of *PvFBA* members further separated the conventional class I members from the atypical *PvFBA8* member (class II). Chloroplast-localized Subfamily I members (PvFBA1–PvFBA4) exhibited higher pI values (6.86–9.04) than cytoplasmic members (PvFBA5–PvFBA7, pI 5.93–7.48), likely reflecting adaptation to the alkaline stroma environment. All Subfamily I members shared similar MW (37–45 kDa) consistent with a conserved glycolytic domain (Table 1 and Figure 2B). A class II FBA (PvFBA8) was identified in seashore paspalum, which contained 1376 aa and 42 exons, a significantly higher number than class I FBAs (PvFBA1–PvFBA7) (Table 1 and Figure 2A). Similar phenomena have also been observed in the *FBA* gene family from other plants, such as rice [37], maize [38], tomato [12], and potato (*Solanum tuberosum* L.) [41]. In addition, PvFBA8 displayed an exceptionally high MW (147.8 kDa) and a slightly positive GRAVY (0.110), suggesting additional structural domains and possible hydrophobic surface patches (Table 1). These results suggest that PvFBA8 may represent a structurally divergent FBA member, although its specific biochemical activity and biological role remain to be experimentally verified.

It is noteworthy that the predicted secondary and tertiary structures of the PvFBA proteins (Figure 3 and Table S4) are highly consistent with the previously reported tertiary structure of the potato FBA proteins [41], supporting the conservation of *FBA* genes during their evolution in vascular plants. In addition, a previous cell-type foundational gene analysis identified *FBA* genes as ultra-conserved core genes in vascular plants [42]. Nevertheless, structural conservation does not necessarily indicate identical physiological function. For example, functional impairment of *OsFBA1* leads to gradual chlorosis at the two-leaf stage and eventual death at the three-leaf stage in rice, whereas the loss of *OsFBA3* [43,44] and *ZmFBA8* [38] function does not affect plant survival. These findings suggest that *OsFBA1* plays a unique and irreplaceable role in sustaining photosynthetic activity and carbon assimilation, while *OsFBA3* and *ZmFBA8* exhibit partial functional redundancy. These observations indicate that *FBA* genes appear to have undergone functional diversification and redundancy during vascular plant evolution.

*Cis*-acting regulatory elements, including enhancers and promoters, play essential roles in plant growth, development, and physiology by flexibly regulating gene expression [45]. In this study, the promoter regions of *PvFBA* genes contained multiple growth/development-, light- and stress-related elements (Figure 4 and Table S3). Most *cis*-acting regulatory elements were phytohormone-responsive (especially abscisic acid and MeJA-responsive elements) and light-responsive, a pattern consistent with observations in potato [41] and wheat [11]. Some elements have been widely reported in other plant species as key mediators of heavy metal detoxification and stress signaling. ABREs are known to integrate abscisic acid signaling under various abiotic stresses, and accumulating evidence suggests that cadmium stress triggers ABA accumulation, which in turn activates downstream defense genes via ABRE-dependent pathways. Similarly, G-box elements serve as binding sites for MYB transcription factors, and recent studies have demonstrated their direct involvement in cadmium tolerance. This result suggests that *PvFBA* gene expression is tightly regulated by abscisic acid and MeJA signaling pathways, which are well documented to mediate plant abiotic stress responses, including cadmium tolerance [46,47]. The promoter regions of some *PvFBA* genes, such as *PvFBA1* and *PvFBA2*, showed a high degree of similarity in *cis*-acting regulatory elements, which was speculated to have a similarity in gene expression. However, because promoter analysis is based on software prediction, the regulatory roles of these elements require further validation, such as promoter-reporter assays or transcription factor binding analyses.

FBAs are key enzymes in plants, participating in numerous critical physiological and biochemical processes, including energy metabolism, signal transduction, plant development, and defense and response to biotic and abiotic stresses [4,7,38,48]. Phylogenetic and synteny analyses (Figure 1 and Figure 5B) revealed that seashore paspalum is more related to maize than to rice. *OsFBA1*, which was essential for regulating chloroplast development, energy generation, photosynthesis, and carbon metabolism in rice, was upregulated by the transcription factor mEmBP-1, which directly binds to the G-box motif in its promoter region [37,49]. MiR775A inhibits the expression of *ZmFBA4*, which leads to the activation of the expression of *ZmFBA8* and the regulation of the glycolytic pathway [50]. PvFBA6 can bind Cd, directly enhancing the phosphorylation level of the PvFBA6 protein, enhancing aldolase activity to increase soluble sugar content and facilitate the pentose phosphate pathway, ultimately boosting NADPH levels for antioxidant synthesis and improving cadmium tolerance in plants [10]. These studies support the biological relevance of *FBA* genes, but the functions of individual *PvFBA* members identified here still require targeted validation.

Although multiple previous studies have identified FBA proteins as cadmium-binding proteins [10,51], the PvFBA members lack classical cadmium-binding motifs (e.g., Cys-rich clusters and Cys/His coordination sites). This suggests that cadmium binding of the FBA protein may occur through non-canonical sites such as the native Zn^2+^ pocket or surface residues. To explore the transcriptional responses of *PvFBA* genes to cadmium tolerance, the public transcriptome data of seashore paspalum [33] was analyzed. Notably, *PvFBA6* and *PvFBA7* showed relatively high expression levels in leaves, with expression trends of initial decrease, followed by increase, and subsequent decrease (Figure 6), highlighting their potential key roles in mediating cadmium tolerance. Notably, *PvFBA2* and *PvFBA3*, as well as *PvFBA6* and *PvFBA7*, which had the most closely related evolutionary relationship (Figure 1), also belonged to the same phylogenetic cluster in the expression levels of *PvFBA* genes. These observations suggest that *PvFBA7*, like *PvFBA6*, is likely involved in regulating cadmium stress responses, warranting further functional characterization. Furthermore, *PvFBA2*, *PvFBA3* and *PvFBA5* showed relatively low expression in leaves, suggesting that they may be predominantly expressed in other tissues or under cadmium stress conditions. These results potentially indicated functional correlation or synergistic action within the relatively close evolutionary relationships of the *PvFBA* gene family.

Several limitations should be acknowledged. First, this study is primarily based on genome-wide identification, in silico analyses, transcriptome mining, and RT-qPCR validation of selected genes; therefore, it cannot establish causality. Second, only one cadmium concentration (300 μM CdCl_2_) and a limited set of sampling time points were examined, which restricts assessment of dose-dependent and long-term responses. Third, only *PvFBA5* and *PvFBA6* were selected for RT-qPCR validation, and additional *PvFBA* members should be validated across more tissues and stress conditions. Fourth, protein-level evidence, including enzyme activity assays, protein abundance, subcellular localization, phosphorylation status, cadmium-binding assays, and protein–protein interactions, is still lacking.

Future work employing functional assays, such as gene knockout, overexpression systems, and protein-level validation, will be necessary to elucidate the proposed roles of *PvFBA* genes in cadmium stress. In the future, integrating transcriptomics, proteomics, and metabolomics data could provide a more comprehensive understanding of how FBA proteins regulate plant responses to environmental stress, thus paving the way for the development of cadmium-tolerant plants.

## 5. Conclusions

In this study, seven class I and one class II *PvFBA* genes were systematically identified for the first time from the whole genome of seashore paspalum. The physicochemical properties, evolutionary relationship, gene structure, conserved domain, protein structure, *cis*-acting regulatory elements, chromosomal localization, and collinearity relationship of eight *PvFBA* genes were analyzed to reveal eight distinct family members with conserved evolutionary and functional characteristics. *PvFBA5* and *PvFBA6* may play essential roles in responses to cadmium stress. These results suggested that *PvFBA* genes played an important role in the response to cadmium stress. This study provided valuable insights for understanding the structure and function of the *PvFBA* gene family, and identified candidate genes for the practical application of cadmium-tolerant plant improvement.

## Figures and Tables

**Table 1:** Characteristics of *PvFBA* genes.

Gene Name	Gene ID in Assembly	Subfamily	Chromosome Location	Protein Length (aa)	Isoelectric Point (pI)	Molecular Weight (Da)	Grand Average of Hydropathicity (GRAVY)	Predicted Subcellular Localization
*PvFBA1*	Pavag08G041800.1	I	Chr08:5925129:5926937:−	388	6.86	41703.57	−0.157	Chloroplast
*PvFBA2*	Pavag05G075100.1	I	Chr05:8468486:8471632:+	386	6.92	41730.68	−0.148	Chloroplast
*PvFBA3*	PavagK331800.1	I	scaffold_906:81050:84461:+	408	9.02	44914.39	−0.218	Chloroplast
*PvFBA4*	Pavag03G086200.1	I	Chr03:7200541:7204303:+	387	9.04	41722.61	−0.218	Chloroplast
*PvFBA5*	Pavag10G188000.1	I	Chr10:36049092:36051598:+	359	5.93	37992.35	−0.053	Cytoplasm
*PvFBA6*	Pavag03G372400.1	I	Chr03:44289962:44292607:−	363	7.48	39466.17	−0.241	Cytoplasm
*PvFBA7*	Pavag03G372200.1	I	Chr03:44252870:44255271:−	363	6.99	39433.10	−0.238	Cytoplasm
*PvFBA8*	Pavag10G109200.1	II	Chr10:10033125:10063220:+	1376	5.98	147793.06	0.110	Cytoplasm

## Data Availability

The original contributions presented in this study are included in the article/Supplementary Materials. Further inquiries can be directed to the corresponding author.

## Supplementary Materials

The following supporting information can be downloaded at https://www.mdpi.com/article/10.3390/cimb48060563/s1.
