# Supplementary material for: Genome-Wide Identification and Expression Analysis of the Fructose 1,6-Bisphosphate Aldolase (FBA) Gene Family Members in Seashore Paspalum in Response to Cadmium Stress
Source: Curr Issues Mol Biol. 2026 May 28;48(6):563. doi: 10.3390/cimb48060563 (PMC13298269; doi:10.3390/cimb48060563)
Supplement: Supplementary file 1 [file cimb-48-00563-s001.zip › Figure S1. The melting curves of PvU2AF, PvFBA5, and PvFBA6 by RT-qPCR.pdf]

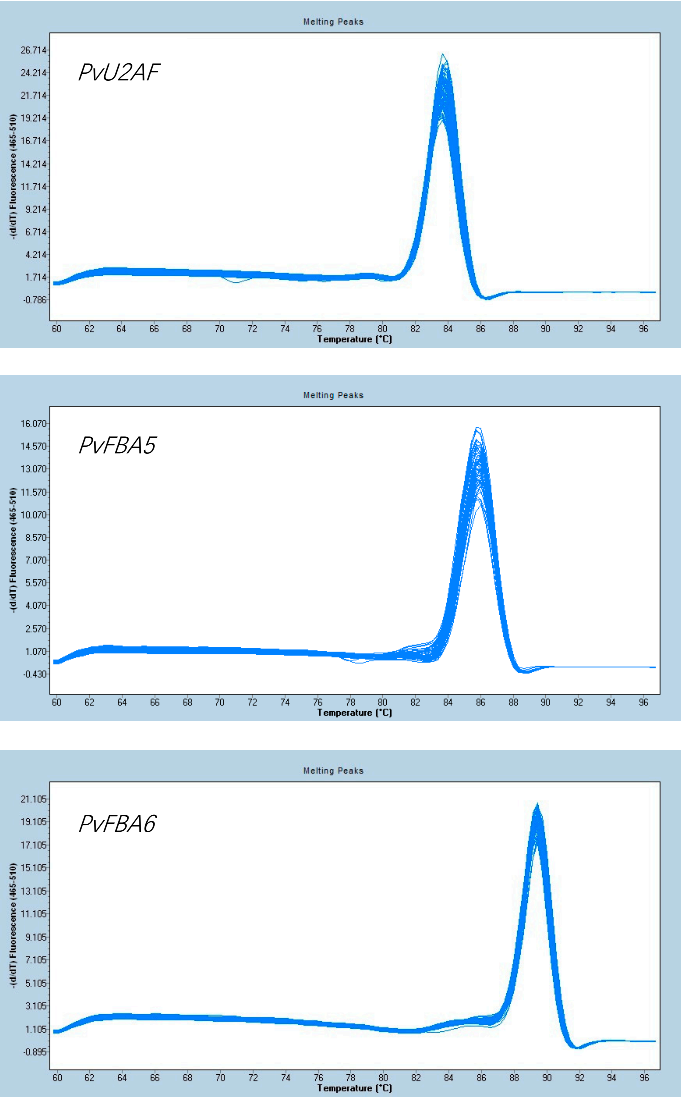

Figure S1. The melting curves of *PvU2AF*, *PvFBA5*, and *PvFBA6* by RT-qPCR. The graph shows RT-qPCR reactions as the negative first derivative of fluorescence with respect to temperature (-dRFU/dT) plotted against temperature, reflecting the decrease in SYBR Green I fluorescence as temperature increases.
