# Supplementary material for: Genome-Wide Identification and Expression Analysis of the Fructose 1,6-Bisphosphate Aldolase (FBA) Gene Family Members in Seashore Paspalum in Response to Cadmium Stress
Source: Curr Issues Mol Biol. 2026 May 28;48(6):563. doi: 10.3390/cimb48060563 (PMC13298269; doi:10.3390/cimb48060563)
Supplement: Supplementary file 1 [file cimb-48-00563-s001.zip › Figure S2. Predicted tertiary structures of PvFBA proteins..pdf]

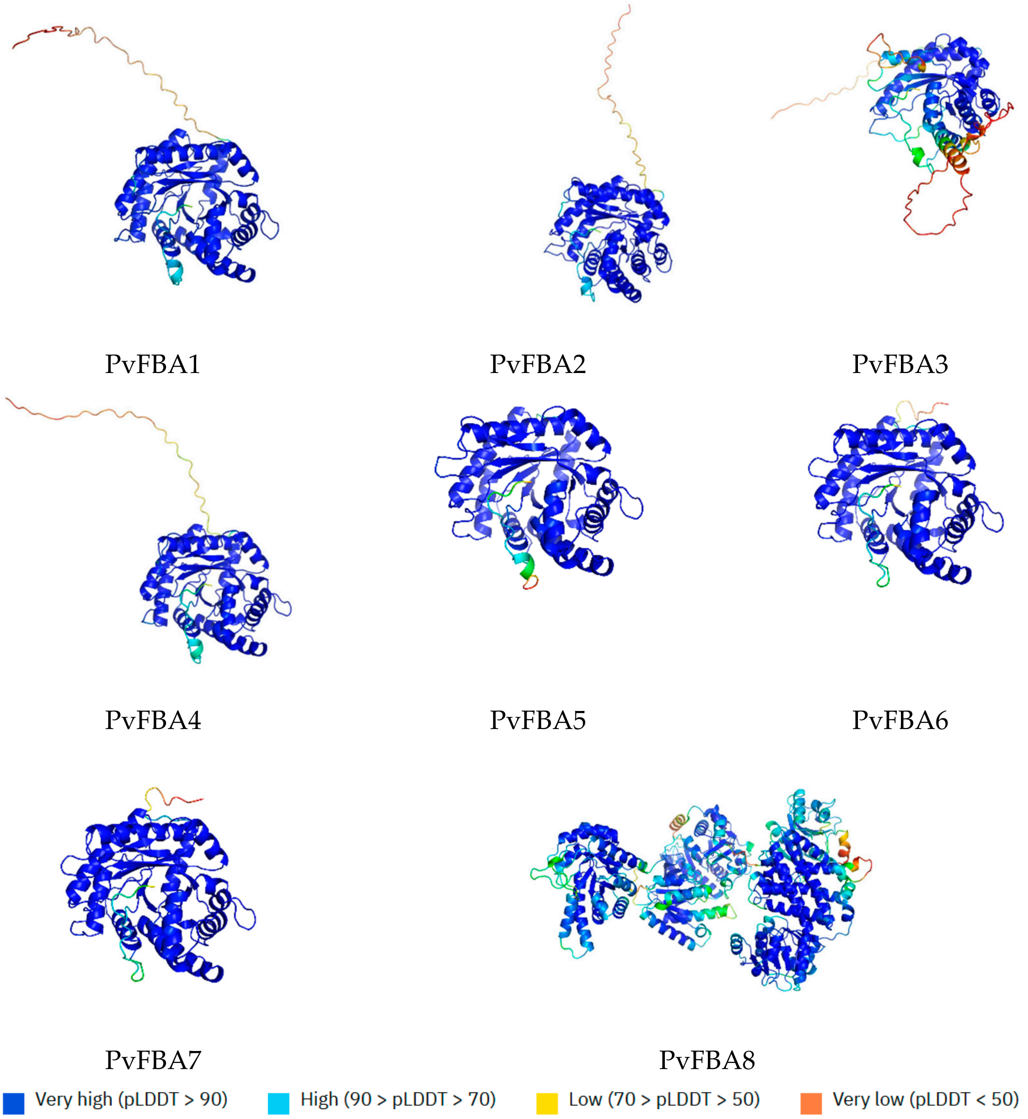

Figure S2. Predicted tertiary structures of PvFBA proteins. The colors indicate per-residue confidence scores (pLDDT), with blue colors representing high pLDDT value and yellow/orange colors representing low pLDDT value.
